# Shared Pleasure in Early Mother‐Infant Interactions Predicts Infant Attachment Security: A Brief Report

**DOI:** 10.1111/infa.70066

**Published:** 2026-01-11

**Authors:** Ida Egmose, Emilie Klein, Cecilie Arentz Munch, Anne C. Stuart, Johanne Smith‐Nielsen, Mette Væver

**Affiliations:** ^1^ Centre of Excellence in Early Intervention and Family Studies Department of Psychology University of Copenhagen Copenhagen Denmark; ^2^ Department of Educational and Psychological Services Hvidovre Denmark; ^3^ Department of Psychology University of Copenhagen Copenhagen Denmark; ^4^ National Institute of Public Health University of Southern Denmark Copenhagen Denmark

## Abstract

Sharing emotions is a fundamental aspect of human interactions. Shared pleasure refers to moments where caregiver and infant engage in mutual smiling while making eye contact. This study examined shared pleasure in early mother‐infant interactions as a predictor of infant attachment security. Associations between shared pleasure and maternal depressive symptoms were also investigated. The sample included 67 mother‐infant dyads, of whom 22 mothers were diagnosed with postpartum depression. Shared pleasure was assessed through micro‐coding of 3‐min mother‐infant interactions recorded in the laboratory at 4 months. Infant attachment security was measured using the Strange Situation Procedure at 13 months. Generalized linear models showed that the mean duration of shared pleasure moments, but not their mere presence, was associated with infant attachment security, with a medium‐sized effect. Maternal depressive symptoms were not associated with shared pleasure. These findings suggest that shared pleasure may be an important early interaction dynamic to foster the development of secure attachment relationships.

## 1 Introduction

The quality of the infant‐caregiver attachment relationship is a well‐established predictor of child socioemotional development (Groh et al. 2017). This relationship develops during the first year of life through ongoing caregiver‐infant interactions in which the sharing and regulation of emotions are central. Therefore, shared pleasure (SP), moments when a caregiver and infant smile or laugh while maintaining eye contact (Mäntymaa et al. 2015), may influence the development of the attachment relationship. This study examines whether SP in early mother‐infant interactions is associated with later infant attachment security. Given that maternal postpartum depression has been associated with disruptions in early interactions (Goodman 2007), we also examine whether maternal depressive symptoms are related to SP.

While caregiver sensitivity is a well‐established predictor of infant attachment security, it does not fully explain its development (Madigan et al. 2024). To support the formation of secure infant‐caregiver attachment relationships, it is essential to identify additional early interaction patterns predicting infant attachment outcomes. Attachment research has largely focused on the regulation of negative emotions, such as caregiver sensitivity to distress (Leerkes 2011). However, because positive and negative emotions may independently contribute to child development (Dallaire et al. 2006), positive emotional exchanges like SP may play a distinct role in forming the attachment relationship.

Previous research has yielded mixed findings regarding the role of SP in child development. In a Finnish sample, longer mean durations of SP moments at 2 months predicted fewer internalizing and externalizing problems at 2 years, buffering the negative impact of parental psychopathology (Mäntymaa et al. 2015). In contrast, a South African study found no associations between SP at 3.5 months and later child cognitive, motor, or language development (Lachman et al. 2022). No prior studies have examined whether SP is associated with infant attachment security. Interestingly, a previous study found shared positive affect in parent‐infant interactions at 7 months to predict infant attachment security at 15 months (Bendel‐Stenzel et al. 2022). However, shared positive affect was defined as simultaneous facial, vocal or bodily positive affect, whereas SP focuses specifically on positive facial affect paired with eye contact.

The relationship between parental psychopathology and SP also remains unclear. In low‐risk samples, one study found higher levels of maternal depressive symptoms to be associated with a higher frequency, but a shorter mean duration of SP moments at 7 months (Puura et al. 2019), while another study found no associations between maternal depressive symptoms and either presence or frequency of SP at 3.5 months (Lachman et al. 2022). Finally, in a high‐risk sample including mothers with a broader range of mental illnesses (e.g., schizophrenia, bipolar disorder, and depression), there was a lower frequency of SP in dyads where mothers had a mental illness (Lachman et al. 2019).

We hypothesize that both the presence and longer duration of SP moments during mother‐infant interactions at 4 months will be associated with greater infant attachment security at 13 months. Given the mixed findings on associations between maternal psychopathology and SP, we explore whether maternal depressive symptoms are associated with the likelihood and duration of SP moments.

## 2 Methods

### 2.1 Participants

The study included 67 mother‐infant dyads, with 24 mothers diagnosed with postpartum depression (PPD). Mothers were on average 31 years old (SD = 4.1), most were of Danish origin (*n* = 62, 92.5%), held at least a bachelor's degree (*n* = 57, 85.1%), and lived with the child's father (*n* = 64, 95.5%). Around half of the infants were girls (*n* = 35, 52.2%). There were no significant demographic differences between mothers with and without postpartum depression (*p*s ≥ 0.233), suggesting a generally resourceful sample aside from maternal depression.

Nonclinical mothers were recruited during their third trimester via advertisements on maternity‐related websites and at local obstetricians. Mothers with postpartum depression were referred by their health visitor between 8 and 14 weeks postpartum if they scored 10 or more on the Edinburgh Postnatal Depression Scale (EPDS; Cox et al. 1987).

Inclusion criteria were first‐time motherhood, singleton pregnancy and for the PPD group fulfillment of diagnostic criteria for a postpartum depression. Exclusion criteria were substance abuse, psychotic symptoms, severe medical conditions in the mother within 1‐year postpartum, premature birth, and major child disabilities.

The study was conducted in accordance with the Declaration of Helsinki and received approval from the departmental ethical review board (approval no. IP‐IRB 2014/02). Written informed consent was obtained from all mothers on behalf of themselves and their child prior to data collection.

### 2.2 Procedure

Mothers in the clinical group were interviewed using the Present State Examination (PSE; Wing et al. 2012) after referral to confirm PPD according to the Diagnostic and Statistical Manual of Mental Disorders (DSM‐V‐TR). Mothers in the nonclinical group were assessed using the PSE upon enrollment in pregnancy to confirm absence of a mental disorder, and their EPDS scores were collected at 6–8 weeks postpartum. Sociodemographic data were collected via questionnaire.

At 4 months, dyads participated in a 10‐min face‐to‐face free‐play interaction video‐recorded in the university lab. At 13 months, dyads returned to the lab for the Strange Situation Procedure (SSP).

### 2.3 Measures

#### 2.3.1 Shared Pleasure

A three‐minute segment from each interaction was micro‐coded using Beebe et al. ’s (2010) coding systems. All coding schemes included a “non‐codable” category, applied when coding was not possible due to visual occlusion of the infant's or mother's face. An independent coder double‐coded a randomly selected subset of the interactions to assess interrater reliability.

*Infant Gaze* was coded frame‐by‐frame as either directed toward or away from the mother's face. Gaze shifts were coded if sustained for min. 80 milliseconds. Interrater reliability was moderate to strong calculated from 1 minute of each interaction (time‐based *κ* = 0.88, event‐based *κ* = 0.68; Bakeman and Quera 2011).

*Infant Facial Affect* was coded frame‐by‐frame using five categories: high negative, mild negative, neutral/interest, low positive, and high positive. Shifts in facial affect were coded if sustained for min. 280 milliseconds. Interrater reliability was moderate to strong calculated from 29% of the interactions (time‐based *κ* = 0.82, event‐based *κ* = 0.63; Bakeman and Quera 2011).

*Maternal Facial Affect* was coded second‐by‐second using eight categories: mock surprise, positive face (smile), interest, blank, woe face, negative face, threat face, and turned away from baby. Thus, in all other codes than “turned away from baby,” the mother was oriented toward the baby. Interrater reliability was moderate calculated from 20% of the interactions (*κ* = 0.72).

*Shared Pleasure* was identified as instances where (a) the infant gazed at the mother, (b) the infant displayed positive facial affect (low or high), and (c) the mother displayed positive facial affect (mock surprise or positive face). Infant gaze and facial affect were recoded to second‐by‐second to match the temporal resolution of maternal facial affect.

#### 2.3.2 Infant Attachment Security

The Strange Situation Procedure (SSP; Ainsworth et al. 1978) was used to assess the quality of the infant‐mother attachment relationship. We used both a dichotomous measure of infant attachment security (secure vs. resistant or avoidant) and a continuous measure (Richters et al. 1988; Van Ijzendoorn and Kroonenberg 1990), with higher scores indicating greater attachment security.

All SSPs were coded by a primary coder. A second coder independently coded a randomly selected subset (23%) resulting in moderate interrater reliability (*κ* = 0.59). Both coders passed the Minnesota reliability test.

#### 2.3.3 Maternal Depressive Symptoms

The Edinburgh Postnatal Depression Scale (EPDS; Cox et al. 1987) was used to assess the presence of maternal depressive symptoms in the past 7 days. The scale consists of 10 items rated on a scale from “No, not at all” (= 0) to “Yes, quite a lot” (= 3). The EPDS has been demonstrated to be a valid measure of maternal postpartum depression (Smith‐Nielsen et al. 2018).

### 2.4 Statistical Analyses

We used generalized binary logistic and linear models to test hypotheses for dichotomous and continuous outcomes, respectively. The first two models examined the presence (model 1) and mean duration of SP moments (model 2) as outcomes with maternal depressive symptoms as main effect. Maternal educational level in years and infant sex were covariates. The next two models examined the dichotomous (model 3) and continuous (model 4) measures of infant‐mother attachment security as outcomes with presence and mean duration of SP moments as main effects. Maternal educational level in years, maternal depressive symptoms, and infant sex were covariates. Significance was evaluated at *p* < 0.05 (two‐tailed). Since an a priori power analysis was not conducted, we did a post hoc power calculation to determine the sample size required to detect a significant effect of SP on attachment based on our results using G*Power (Faul et al. 2007).

## 3 Results

Most dyads had at least one SP moment (*n* = 58, 86.6%). Among these, the mean duration of SP moments was 2.0 s (SD = 1.1, range = 1.0–5.7). There were no significant associations between SP and maternal depressive symptoms (Table 1). However, the mean duration of SP moments, but not their mere presence, was significantly associated with the continuous measure of infant attachment security (*β* = 0.30, *p* = 0.035), indicating a medium‐sized association. There were no significant associations between the presence of SP moments and infant attachment security (Table 2).

**TABLE 1:** Parameter estimates for predictors of SP.

	SP mean duration						SP presence					
	*b*	*β*	95% CI		SE	*p*	*b*	Odds ratio (exp (*b*))	95% CI		SE	*p*
			LL	UL					LL	UL		
Maternal depressive symptoms	< −0.01	< −0.01	−0.25	0.24	0.02	0.975	−0.08	0.92	0.81	1.05	0.07	0.238
Maternal education (years)	−0.02	−0.3	−0.27	0.22	0.10	0.819	0.28	1.33	0.76	2.34	0.29	0.325
Child sex (reference, boy)	0.19	0.08	−0.17	0.33	0.30	0.525	−0.14	0.87	0.20	3.80	0.75	0.849

Abbreviations: *β*, standardized beta; *b*, unstandardized beta; CI, Confidence interval; LL, Lower limit; SP, Shared pleasure; UL, Upper limit.

**TABLE 2:** Parameter estimates for predictors of infant attachment security.

	Continuous attachment security score						Dichotomous attachment security classification					
	*b*	*β*	95% Wald CI		SE	*p*	*b*	Odds ratio (exp (*b*))	95% Wald CI		SE	*p*
			LL	UL					LL	UL		
SP mean duration	0.69	0.30	0.02	0.57	0.33	0.035	0.76	2.15	0.93	4.98	0.43	0.075
SP presence (reference, present)	0.43	0.05	−0.23	0.33	1.16	0.711	0.60	1.83	0.24	13.93	1.04	0.560
Child sex (reference, boy)	−0.30	−0.05	−0.29	0.18	0.66	0.652	0.089	1.09	0.35	3.39	0.58	0.880
Maternal depressive symptoms	−0.09	−0.21	−0.44	0.02	0.05	0.079	< −0.01	1.00	0.92	1.09	0.05	0.996
Maternal education (years)	0.11	0.06	−0.17	0.29	0.22	0.616	< 0.01	1.00	0.68	1.47	0.20	0.997

Abbreviations: *β*, standardized beta; *b*, unstandardized beta; CI, Confidence interval; LL, Lower limit; SP, Shared pleasure; UL, Upper limit.

Aiming for 80% power at a significance level of 5%, a total of 99 participants (continuous attachment security score) and 107 participants (dichotomous attachment security classification) would be needed to detect a medium combined effect of SP presence and SP mean duration, corresponding to Cohen's *f* ^2^ of 0.14 and an odds ratio of 2.7, respectively.

## 4 Discussion

The aim of this study was to examine how SP in mother‐infant interactions at 4 months relate to concurrent maternal depressive symptoms and later infant attachment security. Most dyads (86.6%) had at least one SP moment, similar to most previous studies reporting rates between 71% and 82% (Lachman et al. 2022; Mäntymaa et al. 2015; Puura et al. 2019). An exception is the high‐risk South African sample, where only 20.5% of the dyads had at least one SP moment (Lachman et al. 2019). The mean duration of SP moments was 2 s, similar to the only previous study reporting this metric (Mäntymaa et al. 2015).

Maternal depressive symptoms were not associated with either the presence or mean duration of SP moments, which is in alignment with some previous studies (Lachman et al. 2022; Puura et al. 2019). However, the South African high‐risk sample found SP moments to be less likely to occur when mothers were diagnosed with a mental illness (Lachman et al. 2019). In addition to its distinct cultural context, this sample also differed from the present by including mothers with a broad range of psychiatric diagnoses (e.g., schizophrenia and bipolar disorder). The sample was also characterized by higher levels of contextual risk, for example, over half of the mothers were divorced, separated or single. Taken together, these findings suggest that maternal depressive symptoms alone may not significantly affect SP. However, when the mother's mental illness is more severe or co‐occurs with high contextual risk, SP may be adversely affected.

Longer durations of SP moments, but not their mere presence, were associated with greater infant attachment security at 13 months. The association with the dichotomous measure of attachment security was not significant, possibly due to reduced statistical power compared to the continuous measure. This finding aligns with previous research showing that longer SP moments, rather than their frequency, predicted fewer internalizing and externalizing behavior problems at age 2 (Mäntymaa et al. 2015). Consistent with previous studies, most dyads had at least one SP moment, suggesting that it is common for low‐risk dyads to engage in SP. However, the ability to sustain SP moments appears less common and may require parents to show both strong emotional engagement and attunement to their infants' cues (Puura et al. 2019). Longer SP moments may therefore reflect a stronger emotional connection and attunement, which are not captured by frequency‐based metrics.

It is important to interpret the findings considering the study's limitations. First, SP was calculated by combining separately micro‐coded behaviors rather than coded directly from the interactions as done in previous studies. Further, maternal gaze was not coded separately, as mothers typically looked at their infant. However, turning the face away from the infant was registered in the maternal facial affect coding. These circumstances could affect the construct validity of SP. For instance, the mother could be oriented toward the baby without making eye contact, which could lead to falsely identifying SP moments. However, the descriptive statistics for SP in the current study were broadly comparable to those reported in prior research, which support the validity of the derived measure. Second, only a 3‐min segment from each interaction was micro‐coded due to the time‐intensive nature of this approach. Nevertheless, prior research suggests that thin slices can reliably and validly capture behavior across domains (Murphy and Hall 2021). Third, we did not include any infant‐level predictors of SP, despite SP being a dyadic feature. For example, a previous study found that infant temperament contributed to variation in SP (Puura et al. 2019), highlighting the need for future studies to investigate how infant characteristics contribute to SP. Finally, all participating parents were mothers, mostly of Danish origin and well‐educated, which limits the generalizability of the findings to more diverse populations, including different cultural contexts and father‐infant interactions.

In conclusion, this study found that longer mean durations of SP moments were associated with greater infant attachment security. Hereby, the study highlights SP as an important interaction pattern and as such a promising target for early interventions. However, there were no association between maternal depressive symptoms and SP, suggesting that when contextual risk is low, depressive symptoms may not affect SP.

## Author Contributions

**Ida Egmose:** conceptualization, formal analysis, writing – original draft. **Emilie Klein:** conceptualization, formal analysis, writing – review and editing. **Cecilie Arentz Munch:** conceptualization, formal analysis, writing – review and editing. **Anne C. Stuart:** conceptualization, supervision, writing – review and editing. **Johanne Smith‐Nielsen:** investigation, formal analysis, supervision, writing – review and editing. **Mette Væver:** conceptualization, supervision, funding acquisition, resources, writing – review and editing.

## Ethics Statement

The Institutional Ethical Review Board at the Department of Psychology, University of Copenhagen approved the project (approval number: IP‐IRB/2014/02). The study was conducted in accordance with the 1975 Helsinki Declaration as revised in 2008, as well as national ethical guidelines.

## Conflicts of Interest

The authors declare no conflicts of interest.

## Data Availability

The data supporting the findings of this study are available from the corresponding author upon reasonable request. The dataset is not fully anonymized and therefore cannot be made publicly available to protect participant confidentiality.
